# Supplementary material for: Complexome profiling of the Chlamydomonas psb28 mutant reveals TEF5 as an early PSII assembly factor
Source: Plant Cell. 2025 Mar 17;37(6):koaf055. doi: 10.1093/plcell/koaf055 (PMC12164590; doi:10.1093/plcell/koaf055)
Supplement: koaf055_Supplementary_Data [file koaf055_supplementary_data.zip › psb28_interactive.zip!/plots/intensity/qualityPlots/peptides/_Cre06.g303700.t1.2.html]

### Cre06.g303700.t1.2

Trivial name:   
SeqCoverage [median]: 34.70  
Mass: 12.78 kDa  
  
 

| Sequence | MissedCleavage | Mass [Da] | wtIntensity | muIntensity | Score |
| --- | --- | --- | --- | --- | --- |
| AAPAAAASSSAAK | 0 | 1072.55 | 8976.00 | 7027.84 | 103.31 |
| GFAAADQFTSR | 0 | 1169.55 | 1246.78 | 3775.66 | 117.53 |
| GKGFAAADQFTSR | 1 | 1354.66 | 4141.37 | 5382.94 | 80.90 |
| SSGKEEDPNAVVPLKR | 2 | 1724.91 | 12335.94 | 17461.55 | 100.41 |
|  |  |  | 26700.1 | 33648.0 |  |
